# Impact of hearing impairment on the mental status of the adults and older adults in Jordanian society

**DOI:** 10.1371/journal.pone.0298616

**Published:** 2024-03-04

**Authors:** Safa Alqudah, Margaret Zuriekat, Aya Shatarah

**Affiliations:** 1 Department of Rehabilitation Sciences, Faculty of Applied Medical Sciences, Jordan University of Science and Technology, Irbid, Jordan; 2 Department of Special Surgery, School of Medicine, The University of Jordan & Jordan University Hospital, Amman, Jordan; 3 Bachelor in Speech and Hearing, Jordan University of Science and Technology, Irbid, Jordan; The World Islamic Sciences and Education University, JORDAN

## Abstract

**Background:**

Hearing loss is a common disorder, affecting both children and adults worldwide. Individuals with hearing loss suffer from mental health problems that affect their quality of life.

**Objective:**

This study aimed to investigate the social and emotional consequences of hearing loss in a Jordanian population using Arabic versions of the Hearing Handicap Inventory for Adults (HHIA) and the Hearing Handicap Inventory for the Elderly (HHIE).

**Methods:**

This study included 300 Jordanian participants aged 18–90 years with hearing loss. Each participant underwent a complete audiological evaluation before answering the questionnaires.

**Results:**

The median overall scores of the HHIA and HHIE groups were 39 and 65, respectively. Both HHIA (Cronbach’s alpha = 0.79, p < 0.001) and HHIE (Cronbach’s alpha = 0.78, p < 0.001) were significantly associated with the social, emotional, and overall scores. Compared to the adult group, the median emotional and social scores of the older adults group were significantly higher than the adults group (Z = -4.721, p = 0.001), using the Mann-Whitney test.

**Conclusion:**

The present research revealed that psychological disabilities associated with hearing loss in the adult Jordanian population are more frequent and severe than in other nations. This may be attributed to the lack of awareness of the mental consequences of hearing loss among Jordanian healthcare providers and the public.

## Introduction

Deafness and hearing loss are widespread, and can occur in any region or country [1]. According to the World Health Organization (WHO) [1], there are approximately 466 million deaf and hard-of-hearing people worldwide, with 432 million adults. Furthermore, nearly one-third of people over 65 years have a hearing loss disorder. Hearing impairment poses a threat since it affects numerous essential aspects of life [1]. It has a negative impact on speech development and language acquisition in children. Consequently, the abilities of children in several areas, including social, psychological, and emotional will be delayed.

Furthermore, hearing impairment in children causes some of the most common secondary effects, including behavioral and communication challenges, depression and introversion, and self-esteem issues [2]. Hearing is critical for the academic and lifelong success of children [3]. Adults have reported various psychological symptoms associated with early and late hearing loss. However, people diagnosed with both early-onset and late-onset hearing loss have significant rage, refusal, bleakness, social isolation, and fatigue [2]. Adults with early-onset hearing loss may face psychological issues as they age. This group finds ways to integrate hearing impairment into their personalities in these situations, becoming part of their identity. Consequently, they can develop strategies for managing hearing loss and going about their daily lives [2]. This situation differs in adults with late-onset hearing loss. These individuals have developed personalities that are not associated with hearing loss. Hearing loss affects every aspect of their lives, including their job, family, and lifestyle. Therefore, they often struggle to comprehend their current circumstances, and are devastated by hearing loss. They experience the five stages of grief described by Kubler-Ross [2]: sadness-denial-isolation, anger, bargaining, depression, and acceptance. Given the significance of psychological issues related to hearing loss and the diverse challenges that may arise based on the age of onset, audiologists should meticulously evaluate the psychological traits associated with hearing loss alongside conventional audiological assessments.

Recent research suggests limiting the assessment to traditional methods and incorporating psychosocial measures to accurately evaluate the physical and emotional impacts of hearing loss. Conventional audiological tests include otoscopic examination, pure-tone audiometry (PTA), speech tests, immittance measurements, auditory brainstem response (ABR), and otoacoustic emissions (OAEs). Some popular assessment tools for testing the psychosocial consequences of hearing handicap in adults and older adults include the Self-Assessment of Communication (SAC) test, which is a self-report questionnaire used to test implications of hearing impairment and hearing aids [4], Hearing Handicap Inventory for Adults (HHIA) [5], and Hearing Handicap Inventory for the Elderly (HHIE) [6]. The HHIA and HHIE questionnaires were initially developed in English to assess the psychological impact of hearing loss [5, 6]. The emotional dimension has a total score of 48 points, while the psychological dimension has 52 points. Recent studies by audiologists have identified HHIA and HHIE as two of the most commonly used scales for measuring self-reported handicap [7, 8]; therefore, these scales were chosen as the focus of the current study. Furthermore, their ease, reliability, and validity are essential for their acceptance as self-assessment questionnaires [9]. These scales are valuable to clinicians because they demonstrate the relationship between hearing impairments and psychosocial factors.

Many studies have used HHIA and HHIE tools in various languages and countries to evaluate the psychological effects of hearing loss. For example, the HHIA questionnaire was translated, adapted, and validated in Brazilian Portuguese [10]. This study included 30 individuals with normal hearing aged 20–60 years, and 113 individuals with sensorineural hearing loss (SNHL) aged 21–64 years. They found excellent internal consistency comparable to that of the English versions. However, further research on the convergent validity of this instrument is required. In Japan, Tomioka and group [11] adapted and determined the validity and reliability of the HHIE-Screening (HHIE-S). After analyzing the data of 1,731 participants, it was discovered that the HHIE-S had higher reliability than the single question (SQ) measurement. Indeed, the HHIE-S was more sensitive in detecting hearing loss and more responsive in assessing the effect of hearing impairment on quality of life than the SQ.

Liu X-Y, Han Y, and Yang S-M [12] compared the results of the Mandarin-translated and original versions of the HHIE and HHIE-S and investigated the effect of hearing impairment on the quality of life of Chinese people aged over 80. The study included 84 people aged above 80 and was based on eight factors, including age, noise, diabetes, tumors, ear surgery, infection, cerebrovascular diseases, and the use of ototoxic drugs. Forty-eight participants had no hearing loss symptoms, whereas 36 participants had. Furthermore, it was determined that the Chinese-adapted versions of the HHIE and HHIE-S are appropriate for the Chinese population and are interchangeable. Parthasarathy S. and Mathai JP. [13] have translated the HHIA and HHIE questionnaires into Kannada, a South Indian language. They divided 40 individuals with bilateral SNHL into two groups: "Group I" included 20 adults aged 18–55 years, and "Group II" included 20 older adults aged 65–89 years. The study found a significant positive correlation between hearing thresholds and handicap scores in “Group II”. However, this correlation was not reported in "Group I." The South Indian versions of the HHIA and HHIE were reliable and consistent.

Alqudah and coworkers [14] translated the HHIA and HHIE questionnaires into Arabic. They divided 229 individuals into two groups: 115 people with SNHL aged 30–60 years (subject group), and 114 age-matched controls. Based on the age of the participants, both groups were asked to complete the Arabic versions of the HHIA and HHIE. Cronbach’s alpha and Spearman’s correlation coefficients were used to calculate the internal and test-retest consistency. These results suggested that the Arabic versions of the HHIA and HHIE had a strong capability to differentiate between the emotional states of participants with intact hearing ability and those with hearing loss, in addition to estimating treatment effectiveness. Therefore, the Arabic versions of the HHIA and HHIE, equivalent to the original English versions, are considered highly reliable and validated for assessing the psychological effects of hearing loss in Arabic populations.

People with hearing impairments are more likely to suffer from mental health problems than those with normal hearing [2]. As these problems negatively affect the quality of life, this study aimed to highlight the social and emotional consequences of hearing impairments in the Jordanian population and how these consequences are correlated with the type and severity of diagnosed hearing loss. Considering the negative stigma surrounding psychotherapy in the target society, the psychological impact of hearing deprivation in Jordanian adults remains unclear. Thus, an essential implication of the current research is increasing awareness about the emotional and social challenges of hearing impairment. This will ultimately help develop specialized programs counseling the affected people in Jordan, improving their productivity and social skills, and promoting their identity.

## Methods

### Data collection

A cross-sectional study was conducted from 22 March 2022 to 25 May 2023. The study comprised 300 Jordanian participants above 18 years of age and presented with bilateral hearing loss, ensuring a confidence level of 95% and a margin of error equal to 5%. Inclusion criteria for study enrolment required that participants had hearing loss as their sole disability without concurrent speech, vision, or intellectual disabilities. Additionally, participants were excluded if they met any of the following criteria: constant or intermittent use of an amplification system, poor proficiency in Arabic, the duration of hearing loss < 6 months, the presence of unilateral hearing loss, and other physical and mental conditions that may impact work competence and sociability. Before participating in the study, each participant was required to provide a written informed consent approved by the Institutional Review Board (IRB) of the Jordan University of Science and Technology (2022/72).

The data were randomly collected from different places across Jordan, including 73 participants from the Real Sound Clinic and 50 participants from the Sawt Al Hayah for Hearing Aids in Irbid, 40 participants from Alsabeel Center in Amman, 84 participants from the King Abdullah University Hospital in Al Ramtha, and 53 participants from the Government Jarash Hospital in Jarash. Case history, otoscopy, tympanometry, and pure-tone and speech audiometry were used to confirm the presence and severity of hearing impairment. Subsequently, participants with confirmed hearing loss were asked to complete the Arabic versions of the HHIA or HHIE questionnaires based on age. The questionnaires were conducted under the supervision of a well-trained and qualified assistant who explained them to each participant. Afterward, participants had two options for completing the questionnaires via in-person interviews or the Internet.

### Study tools

The HHIA and HHIE are valuable tools for clinicians because they demonstrate the connection between psychological factors and hearing loss [5, 6]. However, the HHIE was created specifically for and standardized on a sample of individuals aged 64 and older; therefore, its usage is constrained. Therefore, Newman and colleagues [5] modified the HHIE to accommodate younger hard-of-hearing adults (individuals aged < 65 years). The HHIA and HHIE are identical and were developed by Ventry and Weinstein [6]. The alternate questions derived from the HHIE for the HHIA focus on the employment implications of hearing loss, making them more appropriate for younger persons. It has been translated into several languages, including Arabic [12]. It has 25 questions, of which 12 pertain to social and 13 to emotional aspects. The main goal of social subsection was to pinpoint situational challenges and demonstrate how they affected the level of involvement of individuals in certain situations. For instance, “Does a hearing problem cause difficulty when visiting friends, relatives, or neighbors? ". Contrarily, emotional inquiries aim to gather information regarding the attitude and emotional responses of respondents to their auditory deficiency and to understand how other people perceive their hearing impairment. “For example, "Does a hearing issue make you feel depressed?". The response options were yes, sometimes, or no, and one of these options was selected for each question. Yes (4 points), sometimes (2 points), and no response (0 points) had different point values. The total scores in the social and psychological sections indicate the percentage of disability, where 0 indicates no disability and 100 indicates a significant disability. The total scores for the emotional and psychological aspects were 48 and 52, respectively.

### Statistical analysis

After collecting the filled questionnaires, data were analyzed using the Statistical Package for the Social Sciences (SPSS) software. Initially, a normality test, known as the Shapiro-Wilk test, was used to determine whether the data had a normal or abnormal distribution, and based on the results, other statistical tests were selected. Both descriptive and inferential techniques were applied to the data analysis. Descriptive statistics were used to summarize and organize the dataset features. Conversely, infrastatistical tests were used to study the relationship between types (conductive, sensorineural, mixed) and the percentages of hearing loss and the total results of the HHIA and HHIE. Finally, comparative tests were conducted to determine whether the psychological impact of hearing loss varied between the older adult and adult groups. The significance level for each test was set at 0.05 (5%), and all confidence intervals constructed in the study were at the 95% confidence level.

## Results

### Demographic characteristics

A total of 300 individuals with hearing impairment participated in this study. Of these, 138 (46%) were female, and 162 (54%) were male. The median age of participants was 56 years (SD = 20.96). Among the 300 participants, 196 (65.3%) were adults, aged 18–65 years, and completed the HHIA. The remaining participants were older adults, aged 65 or above, and completed the HHIE (Table 1). Of the study participants, 132 (44%) finished their bachelor’s degrees, while the remaining were distributed, as shown in Table 1. Furthermore, 88 (29.3%) participants were non-employees, while 212 (70.7%) were employees (Table 1). Regarding hearing loss, 14 (4.7%) individuals showed conductive hearing loss (CHL), 184 (61.3%) showed SNHL, and 102 (34%) showed mixed hearing loss (MHL). The average hearing loss severity for the participants was 48.7 decibels Hearing Level (dB HL).

**Table 1:** The demographic characteristics of adult and older adult subjects.

Variable	Categories	Number of individuals	Percent %
Handicap scale	Adults	196	65.3
	Older adults	104	34.7
Sex	Men	162	54.0
	Women	138	46.0
Type of HL	SNHL	184	61.3
	CHL	14	4.7
	MHL	102	34.0
Profession	Non-worker	88	29.3
	Health-care	10	3.3
	provider	28	9.3
	Teacher	20	6.7
	Engineer	14	4.7
	Manager	8	2.7
	Fieldworker	12	4.0
	Secretary	14	4.7
	Accountant	4	1.3
	Professor	4	1.3
	Military	64	21.3
	Retired	34	11.3
Educational level	Elementary	24	8.0
	Intermediate	38	12.7
	High school	98	31.3
	Bachelor	132	44.0
	Master	10	3.3
	PhD	2	0.7

### The psychological impact of hearing loss

The Kolmogorov-Smirnov and Shapiro-Wilk tests determined that the data were non-normally distributed (p < 0.05). The median total HHIA and HHIE scores were 39 (SD = 26.56) and 65 (SD = 27.20), respectively. The hearing handicap for the total HHIA score was mild to moderate and severe for HHIE. The social score was 22 (SD = 13.06) for HHIA and 36 (SD = 13.15) for HHIE, indicating mild to moderate hearing handicaps for both groups. The emotional score was 15 (SD = 14.21) for HHIA, suggesting no hearing handicap, while it was 32 (SD = 14.92) for HHIE, indicating a mild to moderate hearing handicap (Table 2).

**Table 2:** Scores of hearing handicap inventory of HHIA and HHIE groups.

Groups	Score for social	Score for emotional	Total score	Degree of handicap
HHIA	22 (SD = 13.06)	15 (SD = 14.21)	39 (SD = 26.56)	Mild to moderate
HHIE	36 (SD = 13.15)	32 (SD = 14.92)	65 (SD = 27.20)	Severe

### Correlation between hearing handicap percentage and HHIA and HHIE

The Cronbach’s alpha for the total score among adults and older adults was 0.79 and 0.78, respectively (Table 3). There was a strong correlation between the social, emotional, and total HHIA scores (Cronbach’s alpha = 0.79, p < 0.001) and HHIE (Cronbach’s alpha = 0.78, p < 0.001). The correlation between the two groups was acceptable regarding the hearing loss percentage, whereas the correlation with hearing loss type was acceptable only in the older adult group. The emotional and social subscale scores were not correlated with the type of hearing loss in HHIA, with coefficients ranging from 0.031–0.07.

**Table 3:** The Cronbach’s alpha values in adults and older adults.

Groups	Social	Emotional	Total	Type of hearing loss	Percentage of hearing loss	p-value
Adults	0.968	0.976	0.79	0.05	0.473	1.000
Older adults	0.964	0.972	0.78	0.387	0.436	1.000

### Differences between HHIA and HHIE

A comparison was conducted using the Mann-Whitney Test; the median value for the total scores in HHIA/HHIE was 44. The median values for the emotional and social scores were significantly higher in the older adult group than in the adult group (Z = -4.721, p < 0.001; Table 4). The results indicated that the older adults had more psychological problems than the adult group. Furthermore, the emotional and social scores in the older adults group were acceptably influenced by the hearing loss type (Cronbach’s alpha = 0.387), but remained unchanged in the adults group (Cronbach’s alpha = 0.05) (Table 3).

**Table 4:** The differences in median values for the total, emotional, and social scores in HHIA/HHIE.

	Median HHIA	Median HHIE	Z	p-value
Social score	22	36	-5.504	p < 0.001
Emotional score	15	32	-3.572	p < 0.001
Total score	39	65	-4.721	p < 0.001

## Discussion

The present study showed a significantly higher prevalence of psychological problems among people aged 65 or above than those below 65 years. The handicap was affected by hearing severity in both groups, whereas the type of hearing loss only affected the older adults group.

The study participants aged 65 or below were combined only from social issues resulting from hearing loss. They reported difficulty speaking on telephone, avoiding communication in groups and attending large events, feeling frustrated when talking to coworkers and clients, struggle listening to TV and radio, and going shopping and to restaurants less often than they like. Besides social hinders accompanied with hearing loss, participants aged above 65 years complained of having emotional distress manifested in the form of feeling embarrassed, nervous, irritable, depressed, and uncomfortable when communicating with people or even talking to friends and relatives.

Regarding the overall HHIA/HHIE score, adults performed better than the older adults in the current research. The scores obtained from Jordanian adults were higher than those of Brazilian-speaking participants (mean for total = 52.2; 10). For Brazilian speakers, the mean social score was similar (mean = 25.9); however, the emotional score was higher (mean = 26.3) than the findings of this research. In the Brazilian Portuguese version of HHIA [10], the Cronbach alpha for the total score was found to be 0.92, which exceeded the threshold established in the present study due to variations in types of hearing impairment experienced by individuals in the two study samples. Additionally, the Cronbach’s alpha of the Jordanian version of the HHIE exceeded the Cronbach’s alpha value of the Japanese version of HHIE-S (0.91) [11], which contributed to the differences between the study tools used, mainly in the number of questions included in the short Japanese version of the HHIE-S and the full Jordanian version of the HHIE.

Regarding the mean HHIE in the Chinese Mandarin population [12], the values were 21.9 and 5.5 for total and emotional scores, respectively, which are less than the scores identified in the current study. Nevertheless, the social scores were similar in the two target populations. The variations in the total and emotional scores for the Chinese and Jordanian versions led to a significant difference between the hearing handicaps reported by the current study participants and those of the Chinese study. The percentage of severe hearing handicaps in the present study was higher than that in the Chinese study (17.9%), although the proportion of mild to moderate handicaps was same in both investigations (25%). Hence, the number of Chinese Mandarin participants with no hearing handicap (57.1%) was higher than that of the Jordanian population. The differences between the two groups could be attributed to the fact that most participants in the Chinese study were urban residents.

The research conducted by Parthasarathy and Mathai [13] identified Cronbach’s alpha values of 0.967 and 0.900 for the English HHIE and English HHIA, respectively, indicating that the questionnaires had good reliability, similar to those used in our study. However, according to the current study, the prevalence of psychological issues was much higher among individuals over 65 years than among those under 65. Lin and group [15] demonstrated that dementia is more likely to affect older people with hearing loss than those with normal hearing. Compared to adults with normal hearing, older individuals with hearing loss experience a faster decline in cognitive skills (including memory and focus). Heine and Browning [16] also reported that people with SNHL, a prevalent form of hearing loss among older adults, experience a high incidence of communication breakdown because any decrement in visual or hearing acuity interferes with understanding spoken messages. Poor psychosocial functioning frequently stems from the inability to improve communication performance [16]. Therefore, older persons with sensory loss often struggle to adapt to their loss and eventually develop lethargy, anxiety, depression, and social unhappiness [16].

In older adults, hearing loss further affects the cortical systems of cognitive control [17]. Aging appears to reduce the ability of individuals to filter out extraneous information from sensory systems besides the auditory signal [18–20], which is correlated with heightened activations in the prefrontal and parietal areas, and reduced suppression of activity in the sensory cortex [21] [22]. For example, during an aural task, the older adults (> 61 years old) simultaneously activated both the visual and auditory cortices, failing to reduce irrelevant visual activity, compared to younger adults (40 years old), who suppressed visual signals [18–20]. Given the correlation between the effects of perceptual decline and aging, older adults may always engage in brain mechanisms that encourage difficult listening, exacerbate the impact of noise, and make listening challenging. Indeed, hearing loss adds to the adverse effects of aging [20].

Burfield and Casey [23] concluded that impaired autonomy (independence and control) jeopardized self-esteem and confidence. They stated that people with sensory loss frequently experience anger and frustration with themselves, and in some circumstances, awkwardness and embarassment due to their condition. These mood swings and depressive episodes are frequently observed in aged and sensory loss individuals and are caused by low self-esteem and displeasure with their situation. Despite its rising incidence among older adults, the impact of dual sensory loss on psychosocial performance has not received much attention in the literature [23]. According to Carabellese and coworkers [24], hearing loss and quality of life are significantly correlated. For instance, vision impairment affects mood and social interactions, and hearing loss is closely associated with independence in daily living tasks. A study by Luey [25] have demonstrated the association between poor psychosocial functioning and sensory loss. People with hearing impairment believe that their social skills are lacking. Therefore, they may feel less confident about themselves if their hearing loss, combined with a substandard coping mechanism, is causing them to fail in their jobs. Additionally, some people are reluctant to view their hearing loss as a problem, which result in their reluctance to seek medical attention. This may result in hard-of-hearing people developing additional degrees of handicap, hence increasing the incidence of psychological problems [26].

The SNHL may extend beyond the effects of effortful listening on the auditory system. The damage may affect hearing acuity and extend to impact balance and postural instability, thereby increasing mental health problems. Recent large-scale studies have confirmed a connection between hearing loss and postural control. Children with SNHL show greater postural control instability than their normal-hearing peers [27–29], potentially due to SNHL-induced injury in the cochlear structures, which can co-occur with vestibular dysfunction since the cochlea and vestibule have the same continuous membranous labyrinth of the inner ear [29, 30]. There is a correlation between balance performance and the degree of hearing loss, impaling whether the hearing loss severity is mild or profound, a deterioration in gait performance is noted. Nevertheless, the children with severe and profound SNHL manifested more static equilibrium instabilities than the children with intact hearing [31, 32]. Though SNHL prevalence increases with aging [33], few studies have observed the balance in older adults with permanent hearing impairment [34–37]. Considering these studies, significant alterations in postural balance were also found in this population because of impaired processing of visual and vestibular information, which requires greater assistance from the auditory system to maintain postural balance [38–40]. The results regarding hearing handicap severity among adults align with the data from other studies on Jordanian participants. A recent study in 2020 showed that students with hearing impairments exhibited a medium level of psychosocial and social issues and average coping strategies. The findings demonstrated that students with hearing loss experience social problems like confronting public situations and a fear of being mistreated by others. It has also been reported that these students complained of psychological concerns like explaining mistakes, a fear of making mistakes, and separation anxiety. The adults participating in the current study also had mild to moderate hearing handicap scores with similar social and emotional problems faced by the hearing-impaired students in Jordan [41].

Overall, the results highlight the psychological impact of hearing impairment, particularly among adults and the older adults. This study contributed to the literature by enhancing the role of psychosocial support in hearing impairment. It compared the psychological needs of adults and the older adults associated with hearing impairment, which was notmentioned in previous studies. The only limitation of this study was that self-reported questionnaires were used to asses the mental aspects of the sample rather than comprehensive psychological assessments. These diagnostic examinations are objective, allowing for the collection of more accurate information on the psychological states of the participants. Future investigations should address the effect of using hearing technology on the psychological therapy of hearing-impaired older adults and adults. Further studies should improve the methods for identifying and assessing negative psychological symptoms associated with hearing loss and attempt to reduce their progression. Ultimately, this will improve the mental health and quality of life of individuals with hearing impairments.

## Conclusion

This study highlighted the link between hearing impairment and poor psychosocial functioning in a Jordanian population aged over 18 years. These results confirm the necessity of prioritizing individuals with age-related hearing loss and providing them with ameliorating treatments, including hearing amplification technology, assistive listening devices, and auditory rehabilitation. Identifying individuals with hearing loss, providing them with appropriate hearing aids or other listening devices, and teaching them coping mechanisms may improve their quality of life. The improvement was shown by reducing the psychological pressures on this group and ensuring their regular participation in social activities.

## Supporting information

S1 Data (XLSX)
