# Correction: Impact of hearing impairment on the mental status of the adults and older adults in Jordanian society

**DOI:** 10.1371/journal.pone.0302416

**Published:** 2024-04-16

**Authors:** Safa Alqudah, Margaret Zuriekat, Aya Shatarah

The funding statement is incorrect. The correct funding statement is as follows: This research was funded by the Jordan University of Science and Technology (Grant No. 20220262). The funder had no role in the study design, data collection and analysis, decision to publish, or preparation of the manuscript.
